# MEDTalks: a student-driven program to enhance undergraduate student understanding and interest in medical schools in Canada

**DOI:** 10.3352/jeehp.2019.16.13

**Published:** 2019-05-22

**Authors:** Jayson Azzi, Dalia Karol, Tayler Bailey, Christopher Jerome Ramnanan

**Affiliations:** 1 Faculty of Medicine, University of Ottawa, Ottawa, ON, Canada; 2 Department of Innovation in Medical Education, University of Ottawa, Ottawa, ON, Canada; Hallym University, Korea

**Keywords:** Medical students, Learning, Curriculum, Feedback, Canada

## Abstract

Given the lack of programs geared towards educating undergraduate students about medical school, the purpose of this study was to evaluate whether a medical student–driven initiative program, MEDTalks, enhanced undergraduate students’ understanding of medical school in Canada and stimulated their interest in pursuing medicine. The MEDTalks program, which ran between January and April 2018 at the University of Ottawa, consisted of 5 teaching sessions, each including large-group lectures, small-group case-based learning, physical skills tutorials, and anatomy lab demonstrations, to mimic the typical medical school curriculum. At the end of the program, undergraduate student learners were invited to complete a feedback questionnaire. Twenty-nine participants provided feedback, of whom 25 reported that MEDTalks allowed them to gain exposure to the University of Ottawa medical program; 27 said that it gave them a greater understanding of the teaching structure; and 25 responded that it increased their interest in attending medical school. The MEDTalks program successfully developed a greater understanding of medical school and helped stimulate interest in pursuing medical studies among undergraduate students.

It has been reported that undergraduate university students applying to medical school may be poorly informed regarding this career path [1]. In fact, few programs exist which introduce undergraduate students to medical school [2-4]. Instead, students rely heavily on unofficial resources such as online discussion forums, which have been widely criticized for disseminating inaccurate information [5]. The University of Ottawa Faculty of Medicine’s Medical Education Interest Group created a pilot teaching program in 2015 known as MEDTalks, wherein undergraduate students are exposed to the medical school curriculum, pedagogical approaches, and student experiences [6]. While this program was initially conceived to provide medical students with early experience as teachers, it is possible that this program could have corollary benefits to undergraduate students in terms of providing insight into medical studies. As such, the purpose of this pilot study was to evaluate whether MEDTalks provided undergraduate students with a greater understanding of medical school and to determine whether MEDTalks stimulated their interest in pursuing medical studies.

The 2018 MEDTalks program consisted of 5 sessions (interviewing skills, the musculoskeletal system, psychiatry, cardiology, and respirology) and ran between January and April 2018. Each session generally consisted of a 1-hour large-group session, split into 3 separate 20-minute lectures, followed by a 90-minute session that combined small-group case-based learning (CBL) with a training exercise on physical examination skills , and finally a 30-minute anatomy laboratory session [6]. These sessions were structured to reflect an abbreviated version of a typical week for local pre-clerkship medical students. The objectives of the lectures, CBL sessions, and physical examinations were derived from the formal medical curriculum objectives, the lecture content was prepared by the medical students delivering the talk, and student volunteers acted as standardized patients during the physical examination sessions.

A call for participation in MEDTalks was distributed to undergraduate students aspiring to pursue a career in medicine between November and December 2017 by their faculties. Invitations were open to students from all programs at the University of Ottawa and Carleton University. Pre-clerkship medical students were recruited to volunteer as teachers. As a prerequisite, medical students were required to attend a faculty-led workshop focused on effective teaching strategies.

Upon completion of the program, undergraduate students who attended any of the five 2018 MEDTalks sessions were invited to complete a voluntary and anonymous feedback survey. The protocol was reviewed by the Ottawa Hospital Research Ethics Board; it was considered a study for the purposes of program evaluation, and thus received exempt status. The survey was disseminated by email. Two subsequent survey reminders were sent to participants, with the option to opt out of the study at any point. The 10-item survey was created and administered using SurveyMonkey and consisted of Likert-scale questions, open-ended questions, and closed-ended questions. The survey results were validated as being from independent participants and then collated, codified and analyzed by the same author using Excel 2010 (Microsoft, Redmond, WA, USA). The raw data are available in Supplement 1.

Thirty-five unique undergraduate students participated in the 2018 program, of whom 29 (83%) responded to the survey. There were 22 female students, and their mean age was 19.5 years (Table 1). Most students were completing their studies at the University of Ottawa and were in their first or second year of studies. Twenty-four students were pursuing a science or health science degree. For 26 participants, this was their first year attending MEDTalks (Table 2). Participants reported mainly attending MEDTalks because of their interest in medicine and to gain exposure to the University of Ottawa medical program. Most participants agreed or strongly agreed with the notions that MEDTalks facilitated networking with medical students, provided exposure to the University of Ottawa medical program, and enhanced understanding of medical school teaching structure and setting. Participants agreed or strongly agreed that they had benefited from the small-group lectures, large-group lectures, and anatomy sessions. Twenty-five participants indicated that MEDTalks increased their interest in attending medical school in the future.

Our results demonstrated that this program did indeed help increase participants’ understanding of medical school and also increased their interest in attending medical school. MEDTalks offered participants the opportunity to consider the impact of medical training in their unique personal context, to network with current medical trainees, and to gain exposure to a medical training program.

Undergraduate medical education differs greatly from undergraduate university education. The transition to medical school may be challenging, leading to academic difficulties, increased stress levels, and other mental health issues, for which medical students are already at an increased risk [7]. Thus, by exposing undergraduate students to a simulated medical school experience, MEDTalks may help educate candidates about the medical school environment.

Studies show that medical school candidates manage their undergraduate years by taking advice from different sources, most often the internet (social media and medical forums), which often leads to misinformation [5]. One solution is providing direct opportunities for undergraduate students to network with medical students. It has been shown that a key component of the effectiveness of these introductory programs in promoting a medical career is their ability to effectively answer students’ questions about the field [3], which MEDTalks actively promotes.

These findings agree with those of a prior study indicating that exposure to interactive medical school–like settings increased students’ interest and motivation to pursue medicine [2]. However, other similar programs failed to demonstrate a change in students’ interest in pursuing a medical career [1,4]. Reasons for this discrepancy may include program design, teaching quality, or the fidelity of the simulated medical school experience. Further research is required to address the differences among these simulated medical school programs.

Limitations to consider include the nature of the participant population (specifically, a small sample size reflecting a single institution). Surveys were only sent to participants after the final MEDTalks session, meaning the participants who attended the final MEDTalks session received the survey immediately, while those who only attended an earlier session would have been surveyed weeks after. Despite our attempt to standardize all sessions, slight discrepancies in exposures, experiences, and teachers could have therefore impacted participant satisfaction. Furthermore, registration was voluntary, so selection bias must be considered, as individuals already interested in medicine were more likely to attend.

While our study only assessed the short-term impact of MEDTalks, we have yet to follow participants to determine the long-term impacts of MEDTalks on interest, admission, or success in medical school. Furthermore, it would be interesting to assess whether a similar framework could be used to enhance medical students’ understanding of and interest in specific residency programs. Finally, long-term follow-up of medical students who participated as teachers in MEDTalks could elucidate whether this program could effectively promote career paths that include medical teaching components.

In conclusion, this study demonstrated that simulated medical school programs like MEDTalks can not only increase undergraduate student interest in medical school, but can also provide networking opportunities and increase knowledge of the medical school environment, prior to undergraduate students applying and formally committing to a career in medicine.

## Figures and Tables

**Table 1.** Characteristics of MEDTalks program participants (n=29)

Characteristic	Value
Participants’ characteristics	
Age (yr)	19.5±1.4
Female gender	22 (76)
University of Ottawa	
University of Ottawa, Science	16 (55)
University of Ottawa, Health Sciences	8 (28)
University of Ottawa, Human Kinetics	2 (7)
University of Ottawa, Arts	1 (3)
Carleton University	
Carleton University, Health Sciences	1 (3)
Carleton University, Sciences	1 (3)
Year of undergraduate studies	
Year 1	13 (45)
Year 2	7 (24)
Year 3	5 (17)
Year 4	3 (10)
Year 5	1 (3)

Values are presented as mean±standard deviation or number (%).

**Table 2.** Post-intervention program feedback from the MEDTalks education program participants (n=29)

Questions	Responses
Is this your first year attending MEDTalks?	
Yes	26 (93)
No	2 (7)
Which MEDTalks sessions did you attend this year?	
Interviewing skills	19 (65)
Musculoskeletal system	16 (55)
Psychiatry	14 (48)
Cardiology	16 (55)
Respirology	14 (14)
I attended MEDTalks...	
...because of my general interest in learning about medicine	
Strongly disagree	0
Disagree	0
Neutral	0
Agree	3 (10)
Strongly agree	26 (90)
...to gain exposure to the University of Ottawa medical program.	
Strongly disagree	0
Disagree	0
Neutral	1 (3)
Agree	7 (24)
Strongly agree	21 (72)
...to network with medical students.	
Strongly disagree	0
Disagree	2 (7)
Neutral	2 (7)
Agree	13 (45)
Strongly agree	12 (41)
...to see if medical school is a good fit for me.	
Strongly disagree	3 (10)
Disagree	3 (10)
Neutral	4 (14)
Agree	10 (34)
Strongly agree	9 (31)
MEDTalks allowed me to…	
…network with medical students.	
Strongly disagree	0
Disagree	2 (7)
Neutral	4 (14)
Agree	15 (52)
Strongly agree	8 (28)
… determine if medical school is ‘right’ for me.	
Strongly disagree	0
Disagree	2 (7)
Neutral	11 (38)
Agree	10 (34)
Strongly agree	6 (21)
… gain exposure to the University of Ottawa medical program.	
Strongly disagree	0
Disagree	0
Neutral	3 (10)
Agree	12 (43)
Strongly agree	13 (46)
This initiative gave me a greater understanding of medical school...	
...curriculum.	
Strongly disagree	1 (3)
Disagree	2 (7)
Neutral	3 (10)
Agree	16 (55)
Strongly agree	7 (24)
...misconceptions	
Strongly disagree	1 (3)
Disagree	3 (10)
Neutral	9 (31)
Agree	12 (41)
Strongly agree	4 (14)
...teaching structure and setting (large and small group classes, laboratory learning, etc.).	
Strongly disagree	0
Disagree	0
Neutral	2 (7)
Agree	13 (45)
Strongly agree	14 (48)
...challenges.	
Strongly disagree	0
Disagree	2 (7)
Neutral	8 (29)
Agree	11 (39)
Strongly agree	7 (25)
MEDTalks has increased my interest in attending medical school in the future.	
Strongly disagree	0
Disagree	0
Neutral	4 (14)
Agree	10 (34)
Strongly agree	15 (52)

Values are presented as number (%).

## Supplementary materials

Supplement 1. Data files are available from https://doi.org/10.7910/DVN/TQLYPF.

Supplement 2. Audio recording of the abstract.
